# Current psychological intervention alternatives for the treatment of paediatric headaches: a narrative review

**DOI:** 10.22514/jofph.2025.002

**Published:** 2025-03-12

**Authors:** Ozan Kayar

**Affiliations:** ^1^Department of Psychology, Çankırı Karatekin University, 18100 Çankırı, Turkey

**Keywords:** Adolescent, Child, Headache, Psychological intervention, Treatment

## Abstract

Headaches are considered as major health problems being common in childhood and adolescence and are debilitating, thus, they lead to poor quality and low performance in all walks of life. Among all types of headaches, episodic migraine and tension-type headache are commonly encountered in the aforementioned phases of life and are likely to have devastating impacts as they become chronic. Stress factors related to school, peers and family, mental problems and traumatic experience may play an essential role in the occurrence of headaches affecting the lives of children and adolescents deleteriously. In this regard, there is unanimity on the most effective treatment of both paediatric and adult headaches through a biopsychosocial approach in which specialists from different fields contribute to the process in a collective manner. There is strong evidence that psychological interventions, which are among the basic elements of holistic treatment, provide relief to patients, especially when applied in combination with pharmacological treatment options in the early period. In general, the research has indicated that such treatments significantly improve the quality of life of children and adolescents suffering from different types of headaches by reducing the frequency, duration and intensity of pain, as well as alleviating the psychological symptoms accompanying the pain. In line with the thorough literature overview, this study aims to shed light on the main goals, domains and scope of application of psychological interventions that are widely applied or considered auspicious in the multidisciplinary treatment of paediatric headache in general terms.

## 1. Introduction

Headache is the most common type of pain in childhood and adolescence [1, 2]. Epidemiological studies point out that the prevalence of paediatric headache, especially migraine and tension-type headache (TTH), has increased significantly and almost doubled in the last 30 years [3, 4]. Although studies have reported higher prevalence rates of TTH compared with migraine, migraine proves to generate more annoyance and unfavourable conditions in day-to-day lives of children and adolescents [5, 6].

As in many other diseases, child and adolescent headaches, especially primary ones, are conditions that should be handled with a biopsychosocial perspective in treatment [7]. Medical interventions for the aforementioned pain types basically include the use of pharmacological agents (analgesics, antimigraine drugs) [8]. On the other hand, as significant number of children and adolescents suffer from headache due to excessive drug use, this very condition tends to trigger the risk of chronic daily headache [9]. In addition, the frequency and severity of headaches are generally correlated with the quality of family and peer relationships, academic performance, physical/social activity level and mental status of children and adolescents [10, 11, 12]. Moreover, factors incorporating behavioral tendencies and lifestyle are likely to set the base for occurrence and increase in the frequency and intensity of headache in these individuals [13]. Therefore, it is important to determine the psychosocial factors that trigger and contribute to headache attacks with a multidimensional evaluation and to focus on these components in the treatment process. Furthermore, in line with the findings of field-related studies, pharmacological methods alone are not sufficient in the treatment of paediatric headaches and that the best results are possible with a combined treatment in which psychological interventions are included at an earlier stage [14, 15, 16].

After decades of several research, the role of mental health status in child and adolescent headaches is more than a mere assumption. In particular, anxiety disorders, depressive disorders and attention deficit hyperactivity disorders are common psychopathologies associated with headaches (especially chronic ones) [11, 17, 18]. Furthermore, recurrent headaches are commonly accompanied by cognitive, emotional and behavioral symptoms which generally do not exceed the threshold of psychopathology, but create an additional life burden on children and adolescents [19]. In addition, having a previous mental disorder may accelerate the process of the emergence or chronicisation of headaches, and an existing headache diagnosis may increase the risk of developing psychopathology in this age group [20]. These findings support the idea that an effective and holistic treatment aiming not only to control headaches in children and adolescents but also to reduce the effects of mental health-related factors from the early period to prevent chronic evolution is mandatory [15].

As there is lack of field-related scientific input and research, the aim of this narrative review is to highlight the importance of diverse psychological interventions to be applied in the treatment of paediatric headaches today; to provide a general framework of the effects of such treatment and how they are applied in practice in line with the literature overview and current know-how. Physicians working in this field and specialists from other related health/medical fields are to be informed about the available treatment options in question and a multidisciplinary approach in treatment is to be promoted as a result of the study.

The study was designed as a narrative review. In particular, the academic search was carried out on PubMed, PsycINFO, and Web of Science in order to have access to the most recent evidence on the psychological interventions in terms of effectiveness. In this process, search terms such as “headache”, “child”, “adolescent”, “psychotherapy” and “psychological treatment” were used as keywords and the searches were limited to the studies published between 2021–2024. In addition, further literature search has been carried out that covered the articles, reviews and academic books having pioneering characteristics. The authors who have made significant contribution to this field are also cited within the scope of this review.

## 2. Contributions of psychological interventions to treatment process and the gains achieved

Psychological interventions play an important role in the treatment of paediatric headaches by creating positive and significant repercussions in multiple domains (Fig. 1). There are many studies indicating that treatments within this scope are particularly effective in reducing basic pain characteristics (frequency, intensity and duration), comorbid psychopathological symptoms, impairment in life functioning or high levels of pain-related disability, and painkiller abuse in children and adolescents diagnosed with headache [7, 15, 21, 22]. As far as the gains are specifically concerned, even though the pharmacological treatment cannot be abandoned in the presence of acute severe migraine attacks, psychological interventions contribute to the fact that a person suffering from attacks is likely to manage the pain effectively and shall not be completely dependent on drugs [23]. In addition, especially the children diagnosed with headache who are unable to receive adequate treatment bear the risk of recurrence of pain and the occurance of other physical and psychiatric symptoms they reach adulthood [24]. The skills acquired through these interventions to effectively cope with psychosocial strains in childhood and adolescence may play an important role in improving the prognosis in adulthood [25].

**Fig. 1.** The major positive effects of psychological interventions on children and adolescents with headache. The downward and upward pointing arrows represent the positive effects of psychological interventions as the factors are reflected in the seesaw pattern (A) and (B).

Psychological interventions provide multiple benefits in treatment. To exemplify, these interventions, most of which can be applied in a group setting, do not involve any risk of side effects, especially when compared with pharmacological treatment alternatives [26]. Moreover, they generally increase adherence to treatment and motivation for recovery, therefore, such treatments enable the healing process or coping mechanism faster and lead to an easier achievement of treatment goals [21]. In addition, successful results obtained through these treatments generally tend to be permanent. As a matter of fact, many of the studies conducted in this context indicate that the curative effects of psychological interventions have both short- and long-term effects in children and adolescents upon the termination of the treatment process [25, 26, 27].

## 3. Current psychological intervention approaches for children and adolescents with headache

Psychological treatments for paediatric headaches are adapted forms of treatment methods developed for various age groups suffering from other types of pain [28], and the number of afore-mentioned treatments has increased significantly over the last three decades (Fig. 2). Among these, there is strong evidence, supported by meta-analyses, towards the efficacy of particularly cognitive-behavioral therapy (CBT), relaxation interventions and biofeedback treatment [22, 26, 29, 30]. Furthermore, although their effectiveness has not been tested as much as the aforementioned treatment methods, recent research draw attention to the fact that acceptance and commitment therapy (ACT) and mindfulness-based interventions are promising for the treatment of cases in this age group [21, 31, 32].

**Fig. 2.** Illustration of current psychological intervention options for the treatment of paediatric headache. MB: Mindfulness-Based.

In most of these interventions, the diagnosis of headache has less importance on treatment procedures, and the applications do not differ substantially between other types of pain or headache syndromes [28]. On the other hand, there are some differences between the interventions in terms of their purpose and scope of use in treatment. For example, CBT and ACT are the psychotherapy approaches on their own with multiple treatment components aiming for change in multiple domains (*e.g.*, cognitive, emotional, behavioral and social aspects of headache) [21]. Interventions such as biofeedback, relaxation and mindfulness-based practices are methods designed to promote improvement in more limited areas and often used together as part of a combined treatment [33]. In addition, the common belief is that interventions in this scope should start in the early stages of treatment for children and adolescents whose headaches are chronic, and who experience high levels of disability as a consequence, and also who have serious psychiatric symptoms. The treatment plan and the selection of the intervention to be applied depend on the age (developmental level), gender, family and cultural background of the patients as well as the nature of specific stress factors (*e.g.*, presence of psychopathology in the parent, exposure to domestic violence or peer bullying at school, *etc.*) and comorbid psychiatric symptoms [5, 28]. In brief, such multiple psychosocial variables are to be taken into consideration by practitioners during clinical assessment.

### 3.1 Cognitive-behavioral therapy

Cognitive behavioral therapy (CBT) is the most widely used psychological intervention for the treatment of paediatric headache, with the highest reported evidence of efficacy [22, 34, 35]. The theoretical basis of this approach evolves around the assumption that thoughts shape emotions and behavior, and thus, the most functional way to reduce or eliminate the patient’s headaches and related psychological symptoms is to change maladaptive thought patterns [36].

CBT includes both preventive and curative cognitive and behavioral interventions to address the comorbid mental disorders in children and adolescents with different types of headaches, as well as the psychosocial factors associated with pain (Table 1) [15, 28]. The cognitive components of CBT generally include psychoeducation about headache and treatment, cognitive restructuring interventions aiming to evaluate pain in more realistic and alternative ways, and teaching cognitive coping techniques (*e.g.*, positive self-talk, distraction, guided imagery) [37, 38]. Behavioral techniques, being fundamental part of the treatment process, are mostly designed in a short and structured format and are generally group-focused, internet-delivered or home-based. They can also be parent-mediated though. Among these, especially progressive muscle relaxation, behavioral activation, activity pacing and differential reinforcement are reported to be useful for children and adolescents with headache in the current literature [5, 21].

**Table 1.** Commonly used CBT techniques in the treatment process of children and adolescents with headache.

Treatment technique		General scope	Objective(s)
Cognitive			
	Psychoeducation about pain and the treatment process	• Introducing the biopsychosocial pain model of headaches to the patient and parent; • Providing a general framework of the basic assumptions and aims of CBT treatment and how the strategies to be addressed in the therapy process work in the context of the patient’s story	Increasing the patient’s or parent’s compliance with treatment and motivation to continue treatment
	Cognitive restructuring	• Identifying and discussing with the patient negative or problematic thoughts (*e.g.*, ruminations, catastrophising) and beliefs that may contribute to worsening pain; • Encouraging the application of cognitive strategies that challenge maladaptive thoughts	Changing unhelpful thought patterns through realistic or functional alternative strategies that reduce pain and accompanying psychological symptoms
	Distraction	• Teaching the patient to direct his/her attention to thoughts, images and actions other than pain in order to change the cognitive focus during a headache attack	Reducing excessive focus on the sensation of pain and increasing participation in important or enjoyable daily activities despite pain
Behavioral			
	Deep breathing/progressive muscle relaxation exercise	• Teaching the patient to exercise the diaphragm muscle while breathing in and out deeply at a slow rate, simultaneously tensing and relaxing the large muscle groups in sequence	Reducing muscle tension and stimulation due to autonomic nervous system activity, which can lead to the appearance/increase of pain or distress
	Lifestyle interventions	• Developing behavioral plans with patients to maintain and implement regular or healthy diet and sleep routines as well as appropriate levels of physical exercise	Regulating the functioning of the autonomic nervous system; encouraging more balanced lifestyle that reduces the likelihood of having a headache attack
	Pacing	• Establishing an appropriate and realistic activity rate (pace) that does not worsen the patient’s pain by including planned rest breaks in the daily schedule; • Gradual integration of daily activities into the patient’s life (*e.g.*, sports, social, *etc.*) that he/she tends to avoid due to headaches	Reducing the likelihood of activity-onset or worsening headache and ensuring the sustainability of daily life functioning

*CBT: Cognitive Behavioral Therapy.*

### 3.2 Relaxation interventions

Muscle tension that occurs simultaneously with mental tension and/or that causes mental strain is common in children and adolescents with headache [39]. Tension-type headaches in particular tend to occur as a result of long term tension in the muscles of face, neck and/or scalp [24]. Furthermore, it is stated that such headaches are likely to arise more frequently in cases of exposure to moderate or high levels of emotional stress leading to an increase in the asymmetry of the masticatory muscles [40, 41]. In order to reduce these conditions, various psychological interventions such as progressive muscle relaxation, autogenic training, guided imagery and deep diaphragmatic breathing are often included in the patient’s treatment plan. These relaxation techniques, entailing both cognitive and behavioral components, can be used separately, in combination, parent-mediated or as a treatment component of a psychotherapy approach (*e.g.*, CBT) [37]. The reason why these techniques are particularly preferred is that they generally contribute to the formation and increase in sense of self-efficacy in the patient. As a matter of fact, self-efficacy, which refers to the belief that a person can control or influence his/her physical and mental functions, is considered as one of the most fundamental change mechanisms in the psychological treatment of paediatric headaches [28]. In addition, these techniques are both easy to learn and easy to apply regardless of the age groups [42].

On the other hand, it has been reported in many studies that relaxation interventions are effective and beneficial reducing the frequency, severity and duration of headaches in children and adolescents as well as drug overuse cases [30, 33, 43]. In addition, the use of these techniques in treatment may include different and specific aims. To illustrate, alleviating or preventing general stress reactions that contribute to the occurrence of headaches in the patient, developing body awareness, reducing the general level of arousal, and providing the patient with the ability to relax certain tense muscles that may have triggered or reinforced the pain can be counted among these aims [44, 45].

Progressive muscle relaxation is one of the first psychological treatment alternatives whose effectiveness has been scrutinized and proven in the management of paediatric headaches [46]. This behavioral relaxation technique, which continues to be widely used today, involves the voluntary slow contraction and relaxation of each muscle group in order to understand the difference between feelings of tension and relaxation [47]. The way the technique is handled in the session is usually standardized, and in this process, the patient is first given a practical training on the content of the exercise. Afterwards, the patient is encouraged to apply the skills acquired in the session outside (especially when he/she has attacks). During the training, the patient is asked to tense a single muscle or muscle group (*e.g.*, hands, feet, head, stomach, *etc.*) for 5–10 seconds while taking a deep diaphragmatic breath, and then try to relax the same muscle or muscle group for 10–20 seconds. In addition, when relaxation in multiple muscles is concerned, a top-down (*e.g.*, head, face, shoulders, back) or bottom-up sequence is usually followed [39, 48].

Guided imagery is another relaxation intervention that is frequently used to help children and adolescents cope more effectively with headache attacks. In this cognitive-based treatment technique, reduction in pain sensitivity is aimed (over-focusing on the sensation of pain) during or just before the attack in the patient, to promote a sense of relaxation through the use of imagination, thus, to help the patient continue daily life activities [21]. The use of this technique in treatment, which is sometimes included in the progressive muscle relaxation exercise, similarly involves training the patient in the session and supporting them to apply what they have learnt outside the session. In this process, the therapist asks the patient to visualize places that make him/her feel calm, safe and peaceful, or life experiences that shall lessen his/her pain (*e.g.*, lying on the beach in a quiet environment, having a warm and sincere conversation with mum, *etc.*) and to direct his/her mind to these images in a focused manner. The patient is also encouraged to take deep diaphragmatic breathing during the imagination in order to reduce the tension of the body muscles and thus facilitate the targeted mental relaxation [49, 50]. In addition, sharing an audio or video recording of the guided imagery performed during the session or a general record created in advance can be functional to help the patient to practice the technique on a daily basis [51].

### 3.3 Biofeedback treatment

Biofeedback is a behavioral intervention approach with a physiological component designed to change the experience of pain and perception in patients with chronic pain and to elicit a sense of personal control over physical, sensory and emotional states [15]. Nowadays, biofeedback methods, which are widely used in the treatment of various types of pain, have been reported to provide many benefits in headache cases of almost all ages. These include positive effects such as alleviating headache frequency, analgesic drug use, depressive symptoms, and increased self-regulation in pain management [15, 52, 53]. In addition, there is some evidence that this treatment technique, when used in combination with other psychological interventions, provides a significant improvement, especially in paediatric migraine cases. Moreover, the aforementioned improvement tends to increase over time [26, 33].

Unlike methods such as CBT and relaxation interventions, which can also be applied in group settings, biofeedback targets a single patient in treatment [38]. Although its applications for preschool children are different than a standard biofeedback treatment developed for older children, some physiological goals are first determined with the patient directly targeting pain exacerbations or emotional responses to pain. Afterwards, some computer-compatible physiological measurement devices are connected to the patient, usually in the area where the patient feels the pain more [53]. Later on, focusing on the set target, the patient sees or hears the changes in his/her physiological processes (*e.g.*, temperature, blood volume, pulse rate, galvanic skin responses, *etc.*) in the course of his/her pain through simultaneously provided feedback in the form of computer-mediated audio or visual signals. In addition, this process is repeated multiple times to further develop the patient’s awareness of the moment when physiological parameters change [38, 54].

The next step in the treatment is to incorporate attempts on self-control into the process. At this stage, the patient is supported to try to control his/her physiological processes by focusing on the headache on the one hand and on the feedback provided through signals on the other. In addition, during these trials, the patient is encouraged to practice various mindfulness and relaxation exercises that are usually taught beforehand [54]. In parallel with the sessions carried out in this context, the patient is expected to be in control of his/her reactions that occur in the body during attack over time, thus he/she shall manage the pain much more effectively [47].

### 3.4 Acceptance and commitment therapy

Acceptance and commitment therapy (ACT) is a form of psychotherapy accepted among third-wave CBT approaches and has been reported to reduce pain intensity, anxiety and depression symptoms, and pain-related disability, especially in adults with chronic pain complaints [55, 56, 57]. In addition, there are some auspicious research findings that ACT may be effective in the treatment of various types of pain, including headaches in adolescents [32, 58, 59]. This approach is stated to be particularly propitious for paediatric pain population who have previously been exposed to multiple treatments but have not achieved significant positive results [51].

ACT promotes the viewpoint that pain and pain-induced deterioration in life functions can be reduced with an acceptance of the reality and favors a flexible attitude instead of avoidance or ignorance and prioritizes “personal values” for a more effective pain management. In this context, mindfulness exercises, value-oriented practices and parent-school supported interventions are generally employed in the treatment to increase psychological flexibility in the patient and to change avoidance or negligence that may reduce quality of life [60]. Alleviation or eradication of pain and associated symptoms are not the ultimate goal of treatment. Instead, in this approach, patients lead their lives in accordance with their values and perceptions without allowing pain to be at the center of life. Thus, stimulating and relaxing activities that are meaningful/valuable for the patient are determined in the therapy and the patient is constantly supported to increase his/her participation in such activities despite the pain [56].

### 3.5 Mindfulness-based interventions

Mindfulness-based interventions (MBIs) include diverse methods with a psycho-educational component of significant potential to be used in the treatment of child and adolescent headaches. The most well-known treatment models of such interventions are mindfulness exercises, mindfulness-based stress reduction training and mindfulness-based cognitive therapy, all of which can be applied in a group setting [61].

MBIs have been proved to be effective in the treatment of migraine in adults while reducing attack frequency, pain catastrophising as well as illness-related functional disability. Additionally, these interventions are known to improve the sense of self-efficacy regarding pain control [62, 63, 64]. However, research on the applicability and effectiveness of MBIs for children and adolescents with headache is relatively limited. Yet, some pilot studies with promising results indicate that there are positive improvements in pain frequency, psychological symptoms and quality of life, especially in adolescents with headache as a result of mindfulness-based intervention [31, 65].

The aspect that distinguishes MBIs from the CBT approach is that thoughts cannot be changed according to the upholders of MBIs. However, patients have control over what they perceive or think and shift their focus in a more constructive way, which leads to a change in their reactions (*e.g.*, physical or psychological) [51]. A typical mindfulness exercise for child and adolescent headaches focuses on breathing calmly and allowing bodily sensations such as pain as well as the disturbing thoughts and feelings to come and go without judgement and/or interference. Simultaneously, the patient is encouraged to direct his/her attention to the present moment, to maintain an attitude of staying in the present, and to notice momentary changes in thoughts and sensations in an emotionally neutral manner [31]. Furthermore, this exercise can also be used for younger children by asking them to focus on the taste, texture and sensation of the candy in their mouths while ignoring all other sensations and thoughts [66].

## 4. Conclusions

This review provides an overview of the general and specific aims, efficacy and application procedures of the main psychological interventions that are among the basic components of the holistic treatment of paediatric headache. In line with the current literature scrutinized and as far as the benefits of multiple domains of the psychological interventions are concerned, this study proves to be highly satisfactory. However, there are also some ongoing challenges, particularly with regard to the accessibility of these treatments.

Treatment based on a biopsychosocial and multidisciplinary approach has become the standard of practice in many adult headache clinics, particularly in countries with well-developed healthcare systems. In these treatment settings, the practitioners of psychological interventions are usually clinical psychologists and, psychiatrists trained to deliver them. Some of these interventions can also be provided by neurologists, algologists, nurses and other allied health professionals as long as they are adequately trained [28]. Such integrated health care practices that include psychological interventions are reported to optimize treatment outcomes and reduce costs [67, 68]. On the other hand, unfortunately, the number of both child and adolescent headache clinics with the aforementioned capacity and the number of competent professionals who are highly interested in the psychological treatment of these cases is quite low worldwide [21, 69]. In brief, the lack of specifically trained health practitioners and well-equipped clinics that merely focus on child and adolescent headaches constitute the biggest obstacle to achieve optimal and permanent results in treatment and to promote access to psychological interventions. Another strain is that high number of parents of children with headache do not have sufficient knowledge about the importance and benefits of these interventions in treatment [70]. With this regard, physicians being the primary referral source in treatment, especially those working in the fields of paediatric neurology and child mental health, are to be familiar with current psychological interventions developed for this population and to encourage families to be open to such treatment options for their children. This review is expected to be a highly informative and practical reference source for the specialists working in the field to boost their interest on these intervention techniques.
